# Long-term cancer risk in heterozygous familial hypercholesterolemia relatives: a 25-year cohort study

**DOI:** 10.1186/s12944-022-01666-2

**Published:** 2022-07-02

**Authors:** Kasper Aalbæk Kjærgaard, Sixten Harborg, Henrik Kjærulf Jensen, Signe Borgquist

**Affiliations:** 1 Department of Oncology, Aarhus University Hospital, Palle Juul-Jensens Boulevard 99, 8200 Aarhus N, Denmark; 2 Department of Cardiology, Aarhus University Hospital, 8200 Aarhus N, Denmark; 3 Department of Clinical Medicine, Aarhus University, 8200 Aarhus N, Denmark

**Keywords:** Cancer, Genetics, Epidemiology, Heterozygous familial hypercholesterolemia

## Abstract

**Background:**

Heterozygous familial hypercholesterolemia (HeFH) due to low-density lipoprotein receptor (LDLR) mutations predisposes patients to highly elevated levels of cholesterol, and patients are at increased risk of adverse cardiovascular events and other morbidities. Whether the *LDLR* mutation and high cholesterol levels affect the risk of cancer remains unknown. The purpose of the present study was to assess the long-term cancer risk in HeFH relatives.

**Methods:**

Study participants were identified by cascade screening during 1992–1994. A comparison cohort was matched 10:1 to the relatives from the Danish general population based on birth year, gender and address. All participants were followed until a cancer diagnosis, migration, death, or end of follow-up as of December 31, 2019. The primary endpoint was any incident cancer diagnosis.

**Results:**

In total, we included 221 relatives with a median age of 37 years (interquartile range: 27–53 years). A total of 117 (53%) of the relatives carried a *LDLR* gene mutation. The crude hazard ratio of our primary endpoint did not reveal any differences in cancer incidence in mutation-carrying relatives compared with the general population cohort (1.18; 95% CI, 0.81–1.71). Nonmutation-carrying relatives however had a lower cancer incidence than the general population (0.45: 95% CI, 0.26–0.80). Thus, the risk among mutation-carrying HeFH relatives compared with nonmutation-carrying HeFH relatives was increased (HR: 2.39; 95% CI, 1.24–4.61).

**Conclusion:**

In Denmark, *LDLR* mutation-carrying HeFH relatives did not have a different cancer risk than the general population. In contrast, nonmutation-carrying relatives had a lower risk of cancer.

## Introduction

Heterozygous familial hypercholesterolemia (HeFH) is an autosomal dominant genetic disorder affecting lipoprotein metabolism in at least 1 in 250 people worldwide [1, 2]. The disease is caused by a mutation in the low-density lipoprotein receptor (LDLR) and leads to impaired clearance of low-density lipoprotein cholesterol (LDL-C) from the circulation and subsequently elevated levels of LDL-C [3]. Untreated HeFH will result in highly elevated levels of plasma LDL-C, which are associated with a substantially increased risk of cardiovascular disease [4–6].

In addition to the risk of cardiovascular diseases, elevated LDL-C levels have further been associated with an increased risk of several types of cancers and cancer progression. [7–12] Given the increased need for cholesterol in proliferating cancer cells, HeFH may be associated with cancer development [7]. The association between HeFH and cancer might be explained by the reported regulatory mechanisms of LDLR levels in plasma e.g. by receptor-mediated endocytosis of serum LDL which enhances the cholesterol level [13]. This is demonstrated in the most common cancers for men and women (prostate cancer and breast cancer, respectively) and in the cancer type with highest mortality rates (lung cancer) [12, 14, 15].

A combined in vivo study on mouse models and publicly available human datasets regarding tumor LDLR expression concluded that high levels of circulating LDL-C and high cellular LDLR expression in cancer cells were associated with high proliferation of estrogen receptor-positive breast cancer cells [15]. Furthermore, low tumor cell LDLR expression was associated with apoptosis and reduced tumor growth in a high-cholesterol setting [15]. Therefore, we hypothesized that elevated LDL-C levels in mutation-carrying HeFH relatives was associated with an increased incidence of cancer.

We aimed to investigate the association of genetically confirmed HeFH among relatives with a predisposed high LDL-C level on the risk of cancer.

## Methods and materials

### Study design and cohorts

**Fig. 1:**
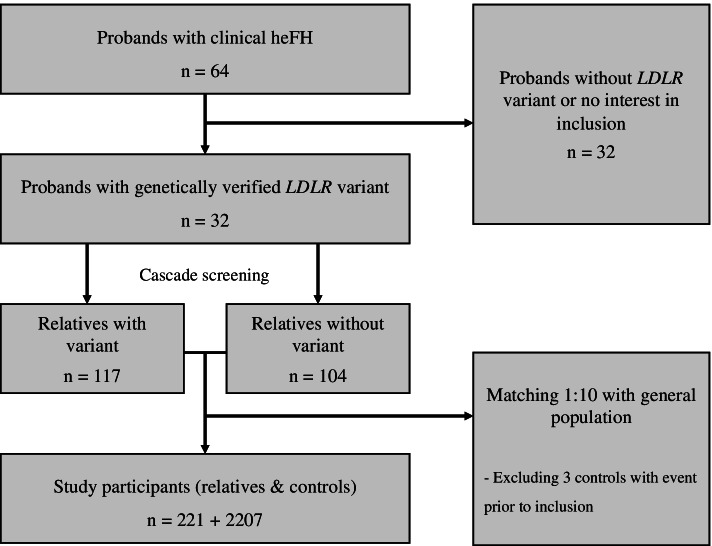
Flowchart with selection of study participants. The process of selecting study participants. HeFH, heterozygous familial hypercholesterolemia; LDLR, low-density lipoprotein recepter

During 1992–1994, patients with prevalent clinical familial hypercholesterolemia (FH) or newly diagnosed FH (probands) were screened and included in a cohort at the “Lipid Clinic” at Aarhus University Hospital, Denmark. HeFH was clinically defined as a plasma level of total cholesterol > 8.0 mmol/L (308.9 mg/dL), LDL-C > 6.0 mmol/L (231.7 mg/dL; if available), presence of tendinous xanthomata in the patient or a first-degree relative, and a family history of hypercholesterolemia. All probands identified were offered genetic testing [16]. Subsequently, cascade screening including genetic testing was performed on all family members if LDLR mutation was detected in the proband, and the mutation was followed as far as possible in the respective family pedigree. Both mutation-carrying and nonmutation-carrying HeFH relatives who thus comprised our study population, were identified (Fig. 1).

At baseline, pretreatment plasma concentrations of total cholesterol, high-density lipoprotein cholesterol and triglycerides were measured by standard enzymatic assays. LDL-C was calculated using the Friedewald Eq [17]. All study participants with confirmed LDLR mutations and thus elevated LDL-C levels were recommended lipid-lowering treatment with statins at study inclusion. Since early 1990s, statins have been available in Denmark and, thus, all subjects with hypercholesterolemia participating in the study were offered drug treatment. At the time of initiation, the most common statins used were lovastatin, pravastatin and simvastatin. Whether a prescription was redeemed was the individual patient’s choice.

Based on the HeFH study cohort, we generated a population-based comparison cohort based on 10:1 matching from the general Danish population. Participants were matched based on birth year, gender, and address using the Danish Civil Registration System, [18] and controls had to be alive when the corresponding relative entered the study. Study participants were excluded if they had a previous or prevalent cancer diagnosis.

### Registries

Using a wide range of nationwide population-based registries, we retrieved all necessary health care information to complete this study. The Danish National Health Service provides tax-supported health care with free access to all public hospitals to all Danish residents. Furthermore, all Danish citizens are assigned a civil personal registration number, a unique identification number that can be used to link registry data [18]. The Danish National Patient Registry (DNPR) has collected data from all nonpsychiatric Danish hospitals since 1977 and from emergency rooms and outpatient clinics since 1995 [19]. The DNPR provides a full overview of all discharges, and diagnoses are included and classified according to the *International Classification of Diseases* (eighth revision until 1994 and 10^th^ revision hereafter). To identify all cancer diagnoses in the study population, we extracted individual medical records on the HeFH families and control cohort from the DNPR. Furthermore, to ensure compliance and adherence to the recommended treatment with lipid-lowering drugs among the mutation-carrying HeFH relatives, we extracted data and information on medication use from the Danish National Prescription Registry (NPR) [20]. The NPR contains nationwide coverage on all prescription drugs redeemed in Denmark since 1994. The Danish Civil Registration System provided information on vital and migration status for each individual in the study cohort during the follow-up period [18].

### Study outcome

In this study, the primary endpoint was cancer incidence, which was defined as the time from inclusion until the earliest occurrence of cancer regardless of type and location. In situ cancers were not included in the primary outcome. As a secondary outcome, we assessed all-cause mortality. Information regarding vital status was retrieved using the Danish Civil Registration and defined as the time from inclusion until death.

### Follow-up and statistical analysis

Follow-up began at an individual’s date of inclusion and was continued until the first event of cancer diagnosis, migration, death, or December 31, 2019, whichever came first. Median follow-up was calculated. In Table 1, baseline data are presented as absolute numbers (percentages) and medians (interquartile ranges [IQRs]) whenever appropriate. Cancer incidence was graphically illustrated by cumulative incidence using the Kaplan–Meier estimator.

**Table 1:** Baseline characteristics of the study participants

	Mutation-carrying HeFH relatives (*n* = 117)	Nonmutation-carrying HeFH relatives (*n* = 104)	General Population Comparison Cohort (*n* = 2,207)	Probands (*n* = 32)
**Sex**				
Male	56 (47.9)	46 (44.2)	1019 (46.1)	14 (43.8)
**Birth-year group, y**				
Before 1930	13 (11.1)	12 (11.5)	249 (11.2)	8 (25.0)
1930–1939	17 (14.5)	12 (11.5)	289 (13.1)	6 (18.8)
1940–1949	13 (11.1)	16 (15.4)	290 (13.1)	7 (21.8)
1950–1959	24 (20.5)	23 (22.1)	470 (21.3)	5 (15.6)
1969–1969	27 (23.1)	22 (21.2)	489 (22.2)	6 (18.8)
After 1969	23 (19.7)	19 (18.3)	420 (19.0)	0 (0)
**Median follow-up time (IQR)**^a^	25.6 (20.3–28.0)	27.0 (24.1–27.3)	26.0 (18.4–27.0)	23.2 (15.9–28.0)
**Median blood levels, mmol/L (IQR)**				
TC	9.67 (8.30–11.1)	5.56 (4.90–6.55)	⋅⋅⋅	11.6 (10.2–12.4)
HDL-C	1.13 (0.96–1.41)	1.32 (1.10–1.61)	⋅⋅⋅	1.28 (0.99–1.54)
LDL-C	7.75 (6.55–9.20)	3.45 (3.00–4.40)	⋅⋅⋅	9.45 (7.90–10.4)
Tg	1.31 (0.98–1.90)	1.20 (0.88–1.84)	⋅⋅⋅	1.20 (1.00–1.80)

Unless otherwise stated, values are reported as absolute numbers (percentages). Lipid levels were obtained at inclusion and prior to initiation of treatment. The conversion of cholesterol and Tg levels to mg/dL was calculated by dividing the values by 0.0259 and 0.0113, respectively

*HeFH* Heterozygous Familial Hypercholesterolemia, *IQR* Interquartile Range, *TC* Total Cholesterol, *HDL-C* High-Density Lipoprotein Cholesterol, *LDL-C* Low-density Lipoprotein Cholesterol, *Tg* Triglycerides

^a^ Time from study inclusion until event of interest, migration, death, or December 31, 2019

We estimated the incidence rates of cancer and used Cox proportional hazard regression models to estimate crude hazard ratios (HRs) with associated 95% confidence intervals (CIs), comparing the rate of incident cancer in mutation-carrying and nonmutation-carrying HeFH relatives with a reference to a general population cohort. A 95% CI was used to determine statistical uncertainty as consistent with best practice in observational epidemiology [21]. To account for possible clustering effects within families, we used robust variance estimation. No other adjustments was made. Using log–log plots, we graphically assessed the proportional hazard assumptions that were not violated. Furthermore, we calculated the prevalence of primary statin prevention across both groups of HeFH relatives.

In sensitivity analyses, non-melanoma skin cancers were excluded from the analysis as to the low risk of spread and mortality of the disease [22].

All statistical analyses were performed using Stata software (Stata v16.1). The follow-up did not involve any contact with study participants or any interventions. Thus, patient consent and approval from the ethics committee were not required according to Danish regulations. The study was approved by the Danish Data Protection Agency and the Central Denmark Region (record no. 1–16-02–381-19).

## Results

The selection of study participants is shown in Fig. 1. In total, we included 221 relatives from 32 families as well as 2,207 controls from the general population, and the median age at inclusion was 37 years (IQR: 27–53 years) (Table 1). The median time of follow-up was 26.0 years (IQR: 18.7–27.3 years), and 527 incident cancers were observed during 54,507 person-years at risk. Among the 221 relatives, 117 (53%) were carrying an LDLR mutation, of which 56 (48%) were men and 61 (52%) were women.

During follow-up, 108 (92%) mutation-carrying HeFH relatives received treatment with a lipid-lowering drug. The majority were treated with statins, and the most frequently prescribed statins were simvastatin and lovastatin. Among nonmutation-carrying HeFH relatives, only 46 (44%) were on lipid-lowering treatment.

**Fig. 2:**
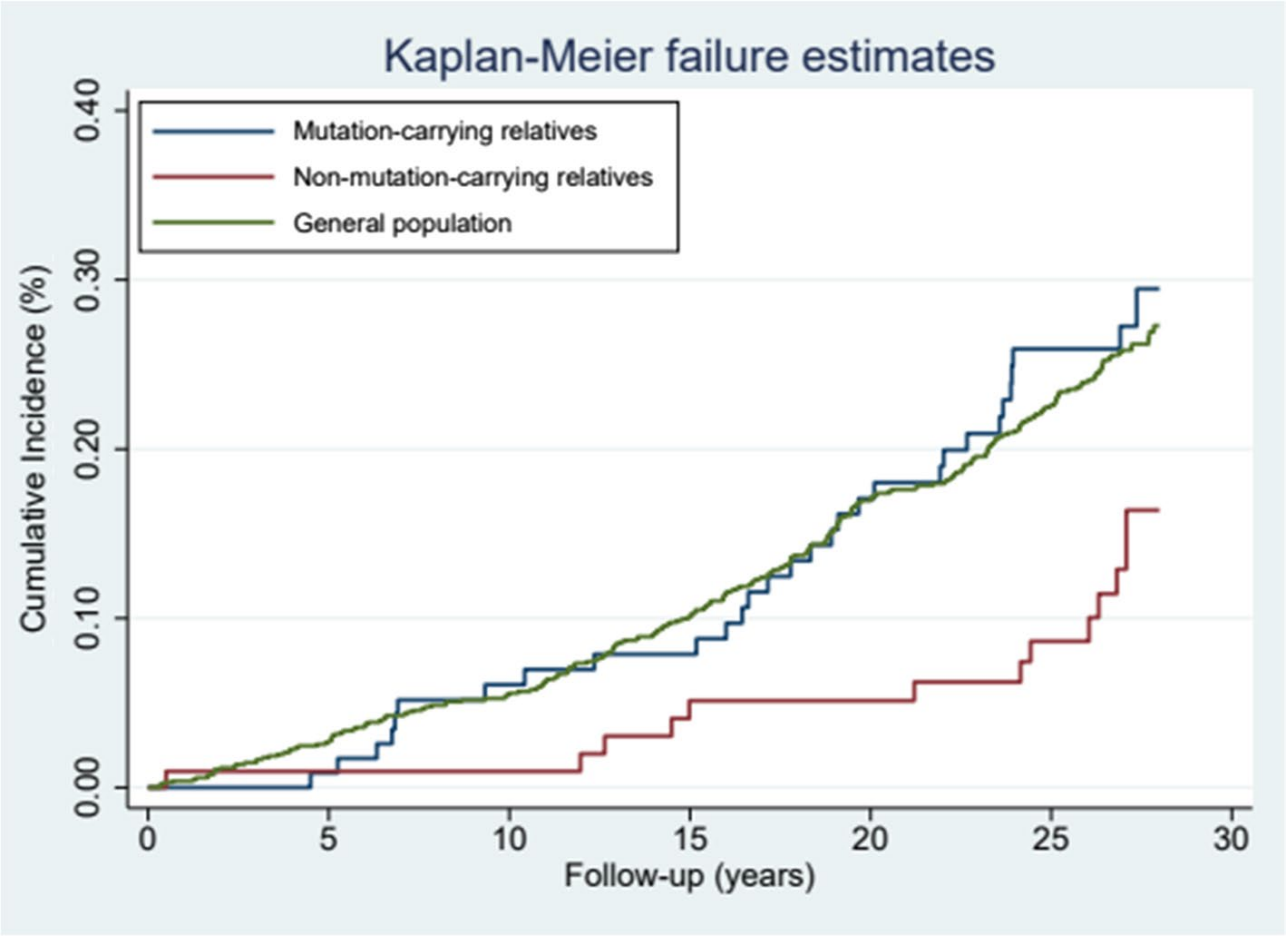
Time to incident cancer. Cumulative incidence illustration of cancer disease in both groups of relatives and the general population

The cumulative incidence of cancer as our primary endpoint is shown in Fig. 2. We observed no difference in cancer risk among mutation-carrying HeFH relatives compared with the general population (HR: 1.18; 95% CI, 0.81–1.71) (Table 2). Among relatives without a mutation, however, we observed a lower cancer incidence compared to controls (HR: 0.45: 95% CI, 0.26–0.80). Thus, the risk among mutation-carrying HeFH relatives compared with nonmutation-carrying HeFH relatives was higher (HR: 2.39; 95% CI, 1.24–4.61).

**Table 2:** Incidence rates and hazard ratios of cancer risk

Exposure of interest	Number of Events	Incidence Rate per 1,000 Person-years (95% CI)	Crude Hazard Ratio (95% CI)
**Any type of cancer**			
General Population (reference)	485	9.96 (9.11–10.9)	1.00
Mutation-carrying HeFH relatives	30	11.3 (7.89–16.1)	1.18 (0.81–1.71)
Nonmutation-carrying HeFH relatives	12	4.81 (2.73–8.47)	0.45 (0.26–0.80)
Comparison of relatives^a^	…	…	2.39 (1.24–4.61)
**All-cause mortality**			
General Population (reference)	563	10.8 (9.98–11.8)	1.00
Mutation-carrying HeFH relatives	22	7.61 (5.01–11.6)	0.70 (0.46–1.07)
Nonmutation-carrying HeFH relatives	18	7.04 (4.44–11.2)	0.65 (0.40–1.03)
Comparison of relatives^a^	…	…	1.08 (0.58–2.02)

*CI* Confidence Interval, *HeFH* Heterozygous Familial Hypercholesterolemia

^a^ Cox regression analyses comparing the two groups of relatives with nonmutation-carrying HeFH relatives as a reference

Examining the outcomes when excluding nonmelanoma skin cancers, the point estimate differed in direction among mutation-carrying HeFH relatives (HR: 0.79; 95% CI 0.45–1.39), whereas the association was similar to the entire cohort among nonmutation-carrying HeFH relatives (HR: 0.39; 95% CI 0.18–0.82) (Table 3). We did not observe any differences in all-cause mortality in any of the groups of HeFH relatives compared with the general population cohort.

**Table 3:** Hazard ratios of cancer risk when excluding nonmelanoma skin cancers

Exposure of interest	Crude Hazard Ratio (95% CI)
**Any type of cancer**	
General population (reference)	1.00
Mutation-carrying HeFH relatives	0.79 (0.45–1.39)
Nonmutation-carrying HeFH relatives	0.39 (0.18–0.82)

*CI* Confidence Interval, *HeFH* Heterozygous Familial Hypercholesterolemia

## Discussion

In HeFH relatives, we assessed the cancer incidence during a long-term follow-up period of greater than 25 years and found no elevated risk of cancer disease among LDLR mutation-carrying HeFH relatives compared with the general population. The risk among HeFH relatives without a genetically confirmed LDLR mutation was significantly lower. Low-malignant cancers, such as nonmelanoma skin cancer, did not drive these results. Despite longer follow-up, all-cause mortality was similar to the previously reported association within this FH cohort [6].

This study is of clinical importance to inform HeFH relatives both with and without an LDLR mutation given that no increased incidence of being diagnosed with cancer has been demonstrated. Furthermore, a lower cancer risk among the relatives without an LDLR mutation was observed. Whether this observation is due to an increased awareness of healthy lifestyle choices or lipid-lowering treatment requires further investigation. The lack of increased cancer incidence is of particular importance, as high levels of LDL-C have been reported to be positively associated with a less favorable prognosis in several major cancer types [23–25].

Our results are similar to that noted among Norwegian FH patients with genetically verified FH [26]. Compared with a matched control group randomly drawn from the general Norwegian population, the incidence of total cancers did not differ. The risk of smoking-related cancers however was associated with a reduced incidence of 20% among the FH patients, most likely due to reduced prevalence of smoking. Unfortunately, we were not able to perform such a stratified analysis in our cohort due to small sample size. Age at HeFH diagnosis/inclusion in the groups of mutation-carrying HeFH patients from both studies was similar (33.7 years in the Norwegian cohort versus 33.0 in our cohort). In our study, however, we were able to follow participants for 26 years (median), whereas median follow-up was restricted to 8.7 years in the Norwegian study.

The recent SAFEHEART study from Spain with more than 3,700 participants (74% mutation carriers and 26% noncarriers) observed that mutation-carrying HeFH relatives have a healthier lifestyle with regard to dietary and smoking habits and physical activity compared with their nonmutation-carrying HeFH relatives [27]. In another Spanish study by Perez-Calahorra et al., the healthy lifestyle among mutation-carrying HeFH patient has been evaluated and compared with a general population cohort [28]. They proved a markedly reduced body mass index and smoking habits in the FH cohort. By assessing these lifestyle habits alone, our mutation-carrying HeFH relatives should expectedly have had a lower risk of developing cancer. However, this notion contradicts the findings of the present study, showing that only relatives without LDLR mutations have a lower cancer incidence than the general population cohort. In the SAFEHEART study, the corresponding relatives with an LDLR mutation had a similar cancer incidence as the background population. Unfortunately, no analyses on differences in lifestyle habits could be performed, as access to relevant lifestyle data was not available in this study.

### Strengths and limitations

This study has some strengths, including the almost complete follow-up of cancer diagnoses during a period of greater than 25 years and the validity of the diagnostic codes used. The HeFH diagnosis is genetically verified. Given that data are obtained from independent medical registries, the results are not biased by self-reporting issues.

In addition, our results should be interpreted within some limitations. Adjustment for lifestyle factors was not possible. Treatment of HeFH includes recommendations toward lowering LDL-C levels by lifestyle interventions, such as a healthy diet, physical activity, discontinuation of smoking, and limitation of alcohol intake; therefore, it would have been valuable to adjust for these factors [29, 30]. However, due to limited data access, this adjustment was not possible. Furthermore, continuous data on LDL-C values would have been of interest to include. Unfortunately, only baseline data were available. Also, the nonmutation-carrying HeFH relatives are expected to be more aware of their own health and lifestyle and thus be more regularly examined by physicians than the general population cohort. This would cause more cancers to be detected and trigger a detection bias, which probably would drive the cancer incidence upward. Thus, this notion cannot explain the lower risk among nonmutation-carrying HeFH relatives. Furthermore, to avoid inducing immortal time bias, we were unable to adjust for the use of lipid-lowering medications. Unfortunately, the small sample size did not allow us to investigate the incidence of individual types of cancers in our HeFH relatives.

## Conclusion

LDLR mutation-carrying HeFH relatives in Denmark were not at increased risk of cancer compared with the general population. In this study, being a nonmutation-carrying HeFH relative was associated with a lower risk of cancer in comparison to the general population. However, these relationships are not casual and to better understand these associations, more research including larger cohorts must be performed, particularly in the context of HeFH relatives without LDLR mutations.

## Data Availability

The data that support the findings of this study are available from the Danish Health Data Authority (Sundhedsdatastyrelsen). Restrictions apply to the availability of these data, which were used under license for this study and so are not publicly available.

## Abbreviations

CI: Confidence interval
DNPR: Danish National Patient Registry
FH: Familial hypercholesterolemia
HeFH: Heterozygous familial hypercholesterolemia
HR: Hazard ratio
IQR: Interquartile ranges
LDL-C: Low-density lipoprotein cholesterol
LDLR: Low-density lipoprotein receptor
NPR: Danish National Prescription Registry
